# Implementation of the Tobacco Tactics intervention versus usual care in Trinity Health community hospitals

**DOI:** 10.1186/s13012-016-0511-6

**Published:** 2016-11-04

**Authors:** Sonia A. Duffy, David L. Ronis, Lee A. Ewing, Andrea H. Waltje, Stephanie V. Hall, Patricia L. Thomas, Christine M. Olree, Kimberly A. Maguire, Lisa Friedman, Sue Klotz, Neil Jordan, Gay L. Landstrom

**Affiliations:** 1 College of Nursing, Ohio State University, Newton Hall, 1585 Neil Ave, Columbus, OH 43210 USA; 2 Veterans Affairs (VA) Center for Clinical Management Research, HSR&D Center of Excellence, 2215 Fuller Road, Ann Arbor, MI 48105 USA; 3 University of Michigan School of Nursing, 400 North Ingalls Building Room 4330, Ann Arbor, MI 48109-0482 USA; 4 VA Center for Clinical Management Research, HSR&D Center of Excellence, 2215 Fuller Road, Ann Arbor, MI 48105 USA; 5 Internal Medicine, Brehm Tower, University of Michigan, Room 6115, 1000 Wall Street, Ann Arbor, MI 48109-5714 USA; 6 Trinity Health, 20555 Victor Parkway, Livonia, MI 48152 USA; 7 The Lacks Cancer Center, Mercy Health Saint Mary’s, 200 Jefferson SE, Grand Rapids, MI 49503 USA; 8 Mercy Health Partners, 1500 Sherman Blvd., Muskegon, MI 49444 USA; 9 Saint Joseph Mercy Health System, 5305 E. Huron River Dr., Ann Arbor, MI 48106-0995 USA; 10 Saint Mary Mercy Hospital, 36475 Five Mile Road, Livonia, MI 48154-1988 USA; 11 Department of Psychiatry and Behavioral Sciences, Northwestern University, Feinberg School of Medicine, Abbott Hall 710 North Lake Shore Drive, Suite 904, Chicago, IL 60611 USA; 12 Center for Management of Complex Chronic Care, Hines VA Hospital, 5000 S 5th Ave., Hines, IL 60141 USA; 13 Dartmouth-Hitchcock Medical Center, One Medical Center Dr., Lebanon, NH 03756 USA

**Keywords:** Smoking, Cessation, Inpatient

## Abstract

**Background:**

Guided by the Reach, Effectiveness, Adoption, Implementation, and Maintenance (RE-AIM) implementation framework, a National Institutes of Health-sponsored study compared the nurse-administered Tobacco Tactics intervention to usual care. A prior paper describes the *effectiveness* of the Tobacco Tactics intervention. This subsequent paper provides data describing the remaining constructs of the RE-AIM framework.

**Methods:**

This pragmatic study used a mixed methods, quasi-experimental design in five Michigan community hospitals of which three received the nurse-administered Tobacco Tactics intervention and two received usual care. Nurses and patients were surveyed pre- and post-intervention.

Measures included *reach* (patient participation rates, characteristics, and receipt of services), *adoption* (nurse participation rates and characteristics), *implementation* (pre-to post-training changes in nurses' attitudes, delivery of services, barriers to implementation, opinions about training, documentation of services, and numbers of volunteer follow-up phone calls), and *maintenance* (continuation of the intervention once the study ended).

**Results:**

*Reach*: Patient participation rates were 71.5 %. Compared to no change in the control sites, there were significant pre- to post-intervention increases in self-reported receipt of print materials in the intervention hospitals (*n* = 1370, *p* < 0.001). *Adoption*: In the intervention hospitals, all targeted units and several non-targeted units participated; 76.0 % (*n* = 1028) of targeted nurses and 317 additional staff participated in the training, and 92.4 % were extremely or somewhat satisfied with the training. *Implementation*: Nurses in the intervention hospitals reported increases in providing advice to quit, counseling, medications, handouts, and DVD (all *p* < 0.05) and reported decreased barriers to implementing smoking cessation services (*p* < 0.001). Qualitative comments were very positive (“user friendly,” “streamlined,” or “saves time”), although problems with showing patients the DVD and charting in the electronic medical record were noted. *Maintenance*: Nurses continued to provide the intervention after the study ended.

**Conclusions:**

Given that nurses represent the largest group of front-line providers, this intervention, which meets Joint Commission guidelines for treating inpatient smokers, has the potential to have a wide reach and to decrease smoking, morbidity, and mortality among inpatient smokers. As we move toward more population-based interventions, the RE-AIM framework is a valuable guide for implementation.

**Trial registration:**

ClinicalTrials.gov, NCT01309217

**Electronic supplementary material:**

The online version of this article (doi:10.1186/s13012-016-0511-6) contains supplementary material, which is available to authorized users.

## Background

Smoking cessation interventions that include counseling, medications, and telephone follow-up for hospitalized smokers have been shown to be efficacious [1]. Hospitalization provides an excellent opportunity for patients to quit smoking because they are a captive audience, are often motivated to quit due to illness, and often quit temporarily due to hospital smoking bans. Moreover, meta-analyses suggest that nurse-administered interventions are efficacious, particularly among hospitalized patients [2]. Despite the strong evidence for the efficacy of inpatient smoking interventions, a large gap exists between the availability of effective smoking cessation interventions and their widespread dissemination in hospitals [3–5]. The challenge rests with disseminating smoking cessation interventions into standard practice.

The *Reach*, *Effectiveness*, *Adoption*, *Implementation*, *and Maintenance* (RE-AIM) framework has been used in other dissemination and implementation health behavior and smoking studies [6–15]. Utilization of the RE-AIM framework is intended to enhance the applicability of research-based interventions in clinical practice and ease the process of planning, conducting, reporting, and selecting interventions to be implemented on a large scale [16]. The constructs of the RE-AIM framework are the following: (1) *reach* (percent and representativeness of individuals receiving an intervention); (2) *effectiveness* (impact of an intervention on outcomes); (3) *adoption* (proportion and representativeness of settings and providers willing to deliver the intervention); (4) *implementation* (extent to which the intervention is implemented as intended); and (5) *maintenance* (sustainability of an intervention at individual and setting levels) [17]. The RE-AIM framework has been used to guide several studies testing the Tobacco Tactics intervention.

A randomized controlled trial (*n* = 184) tested the *efficacy* of the Tobacco Tactics intervention among head and neck cancer patients and found that 6-month smoking cessation rates were 47 % quitting in the Tobacco Tactics group compared to 31 % in the usual care group (*p* < 0.05). Ninety percent of participants said they would recommend the intervention and the manual to someone else dealing with similar issues. As with many efficacy studies, the intervention was not *maintained* and ended when the trial ended [18].

The Tobacco Tactics intervention was then packaged into a toolkit for inpatient nurses and smokers in the Department of Veterans Affairs (VA) [19]. *Reach*: Compared to the usual care site, patients in the intervention sites reported an increase in receiving and satisfaction with selected cessation services, particularly medications (*p* < 0.05) [19]. *Effectiveness*: Six-month quit rates improved from the pre- to the post-intervention time periods in Ann Arbor (*p* = 0.004) and Detroit (*p* < 0.001) (which services a large number of African Americans), compared to the Indianapolis control site (*n* = 1070). *Adoption*: Three hundred sixty-nine or 74 % of targeted nurses and 282 non-targeted personnel were trained in the Tobacco Tactics intervention. *Implementation*: Nurses’ self-reported administration of cessation services increased from 57 % pre- to 86 % post-training (*p* = 0.0002). The intervention was incorporated into new nurse training, and *maintenance* was high as the programs remain in place in Ann Arbor and Detroit 3 years after the study ended [20]. The intervention was exported to another VA via satellite broadcast where it has continued to be implemented 2 years after the study ended [21].

A recently completed NIH-supported R21 randomized controlled trial compared a nurse-supported, web-based version of the Tobacco Tactics intervention versus referral to the 1-800-QUIT-NOW telephone quitline (*N* = 145) among blue collar workers. *Efficacy*: The Tobacco Tactics website group showed significantly higher quit rates (*n* = 18/67, 26.9 %) than the 1-800-QUIT-NOW group (*n* = 6/78, 7.7 %) at 1-month follow-up (*p* = 0.003). *Reach*: Compared to participants in the 1-800-QUIT-NOW group, significantly more of those in the Tobacco Tactics website group participated in the intervention, received phone calls and nicotine replacement therapy (NRT), and found the intervention helpful [22].

These prior studies show that the Tobacco Tactics intervention had high *reach, efficacy/effectiveness, adoption, implementation,* and *maintenance* in VA, non-VA, and community settings. As one of these six studies funded by the National Institutes of Health, which together comprised the Consortium of Hospitals to Advance Research on Tobacco (CHART), this pragmatic trial used the RE-AIM framework [12, 13, 23, 24] to test the nurse-administered Tobacco Tactics intervention on inpatient units in six community hospitals. This study differed from the other CHART trials in that (1) this was a non-randomized, implementation trial while the others were all randomized controlled studies and (2) all but one of the other trials tested variations on referral to quit telephone lines, the other tested referral to website, while this study used real-world staff nurses to deliver the intervention [25–33].

A recently published paper in the *American Journal of Preventive Medicine* describes the *effectiveness* of the Tobacco Tactics CHART study [34]. There were significant improvements in propensity-adjusted, pre- to post-intervention self-reported quit rates and cotinine-verified quit rates in the intervention sites compared to no change in the control sites. Excluding the recently published *effectiveness* results, the specific aims of this study were to

1. determine the *reach* of the intervention by identifying patient participation rates, characteristics of the patient sample, and number of patients that self-reported receiving the intervention;
2. determine the *adoption* of the intervention by describing the units that participated in the study, nurse survey participation rates, characteristics of the nurses, number of nurses trained, and nurses’ opinions about the training;
3. determine the *implementation* of the intervention by describing changes in nurses' attitudes toward providing cessation services, delivery of the components of the intervention, barriers to implementation, documentation of services, and number of volunteer follow-up phone calls made; and
4. determine the *maintenance* of the intervention by evaluating short-term sustainability of the intervention once the study ends.

## Methods

### Design

Details of the study design have been described in a published protocol paper [25]. Using data collected from the larger study, this paper provides information on the process evaluation using the RE-AIM framework, excluding effectiveness (outcome) evaluation, which was recently published separately [34]. Using mixed methods, this quasi-experimental study initially designed to be conducted among a convenience sample of six Michigan Trinity Health community hospitals (matched on size and number of minority patients), of which three were to receive the nurse-administered Tobacco Tactics intervention and three were to receive usual care, although data from one of the control hospitals was not useable due to a protocol deviation. Not to randomize, but only to reduce investigator bias, a random number generator was used to assign the hospitals to experimental and control conditions. While medical surgical units were the primary units targeted, the leaders at the hospitals were allowed to include additional units, which increased the generalizability of study findings. However, nurses and patients on non-targeted units were not followed [25]. Human study approval was received from the University of Michigan (Health Sciences and Behavioral Sciences Institutional Review Board #HUM00043349) and Trinity Health hospitals (Mercy Health Institutional Review Board #2011013, Saint Joseph Mercy Institutional Review Board #HSR-11-1272, and Saint Mary’s Health Care Institutional Review board #SM11-830-01).

### Setting and sample

#### Setting

Trinity Health is one of the largest multi-institutional Catholic health care delivery systems in the nation. Committed to those who are poor and underserved in its communities, Trinity Health serves people and communities in 21 states with 124 continuing care locations, and 91 hospitals, of which five Michigan hospitals were included in the study.

#### Patient sample

Inclusion criteria for the study were patients that (1) smoked a cigarette within 1 month prior to hospitalization, (2) were at least 18 years of age, and (3) had a projected hospital stay of at least 24 h. Excluded were smokers that were (1) involved in a concurrent smoking cessation trial, (2) non-English speaking, or (3) not cognitively or physically able to participate.

#### Nurse sample

Dissemination/implementation research requires attention not only to the individual, but also to the staff and organization delivering the intervention [35]. The nurse sample included nurses from participating inpatient units.

### Procedures

#### Reach and effectiveness

To obtain population quit rates (presented in a prior paper [34]) and patient feedback on tobacco services received, throughout the entire study, all inpatient smokers were identified from the electronic medical record (EMR) and approached by a research assistant to provide written informed consent and a survey. Using a modified Dillman approach, patients were mailed surveys 30 days and 6 months after discharge [36]. Participants were given $10 for each survey returned. At 6 months post-discharge, participants that returned 6-month surveys were provided a urinary NicAlert cotinine test strip to be mailed back, for which they received an additional $20 [37]. Medical information, receipt of cessation services, and quit rates for those lost to follow-up were also downloaded or abstracted from the EMR. Midway through the study, nurses in the intervention hospitals were trained in the Tobacco Tactics intervention, which became the standard of care for treating tobacco dependence for all inpatient smokers in the institution, whether or not they enrolled in the study. In this way, receipt of services and quit rates for all patients were determined pre-intervention, during training (transition period), and post-intervention in both intervention and control groups. Data from patients in the transition period, while nurses were being trained, were not included in the analyses.

#### Adoption, implementation, and maintenance

Nurses in both intervention and control hospitals were anonymously surveyed pre-intervention to assess attitudes about, provision of, and barriers to providing smoking cessation services to inpatient smokers. In the intervention hospitals only, working with the research nurse, master trainers then provided 1-h training sessions on all shifts to targeted nurses who were then immediately surveyed about their opinions about the training. Approximately 3 months after training, nurses in intervention and control hospitals were again surveyed to assess attitudes about, provision of, and barriers to providing smoking cessation services to inpatient smokers. In addition, 10 % of targeted nurses in the intervention sites were interviewed. The training was incorporated into orientation for all new nurses.

### Description of the Tobacco Tactics intervention

#### Tobacco Tactics toolkit for nurses

Based on Agency for Healthcare Research and Quality guidelines, the cessation toolkit included (1) one continuing education unit (CEU) contact hour for training, (2) a PowerPoint presentation on behavioral and pharmaceutical interventions, (3) a pocket card “Helping Smokers Quit: A Guide for Clinicians” developed by the US Department of Health and Human Services, Public Health Service, (4) behavioral and pharmaceutical protocols, and (5) a computerized template for nurse documentation based on the components of Joint Commission (JC) Smoking Cessation standards [38].

#### Tobacco Tactics toolkit for patients

For patients, the cessation toolkit included (1) a brochure, (2) a cessation DVD, (3) the Tobacco Tactics manual [39], (4) a 1-800-QUIT-NOW card, (5) nurse behavioral counseling and pharmaceuticals, (6) physician reminder to offer brief advice to quit coupled with medication sign-off, and (7) follow-up phone calls.

#### Volunteer telephone counseling

When the nurse charted on the documentation template that the patient was given the Tobacco Tactics manual, the EMR was programmed to add the patient’s name and phone number to a list that was forwarded to Voluntary Services two times per week. Trained volunteers at each hospital provided telephone cessation counseling to patients at 2, 7, 14, 21, and 30 days after discharge [20]. Volunteers did not collect research data but did provide documentation that was entered into the EMR.

### Description of usual care

In the Trinity Health System, all inpatients were screened for smoking via the nursing assessment. Nurses were instructed to give smokers brief advice to stop smoking and a brochure.

### Measures

The *reach* of the intervention was measured by calculating patient participation rates and follow-up rates (number of participating/number patients eligible) from recruitment logs. From patient surveys and chart audits, the characteristics of the patient sample were described including demographic characteristics, discharge diagnosis and discharge comorbidity ICD-9 codes categorized according to standard categories [40], the Patient Health Questionnaire 2 (PHQ-2) for depression [41], the Alcohol Use Disorders Identification Test–C (AUDIT-C) for alcohol use [42, 43], and number of cigarettes smoked per day. Differences in pre- to post-intervention patient self-reported receipt of tobacco services were calculated from patient surveys. The *adoption* of the intervention was measured by describing the units that participated in the study and nurse participation rates in pre- and post-intervention surveys and trainings (number of nurses participating/number eligible) from recruitment logs. Pre- and post-intervention nurse surveys provided information on the characteristics of the nurses and nurses’ opinions about the training (overall satisfaction, satisfaction with pharmaceutical management, satisfaction with behavioral management, understanding of training, and helpfulness of training, all of which were rated on a five-point Likert scale).

*Implementation* was measured from nurse surveys by calculating pre- to post-training changes in self-reported attitudes toward providing cessation services, delivery of the components of the intervention, and barriers to implementation. In the intervention sites only, *implementation* was measured via descriptive statistics of documentation of services downloaded from the EMR. In the intervention sites only, interviews were conducted with nurses about their provision of Joint Commission measures for inpatient tobacco cessation (yes/no) and delivery of components of the intervention (yes/no), and nurses were asked to give qualitative comments about their experience implementing the intervention. Volunteer logs revealed the number of phone follow-up attempts, percent reached, average number of calls per patient, number of patients reached, and total number of contacts. *Maintenance* of the intervention was measured by determining whether the implementation continued after the researchers withdrew support from the hospitals as noted in post-intervention patient and nurse surveys (short-term maintenance) and anecdotal communication with nurses after the study ended (long-term maintenance). See Additional files 1, 2, and 3 for details on the measures.

### Data analysis

Descriptive statistics were used to summarize all variables. Patient and nurse pre- to post-intervention differences in the intervention and control sites were compared using chi-square tests of association and *t* tests. The significance level was set at *α* = 0.05, and a two-tailed test was conducted. All analyses were conducted using SPSS version 21 software.

Qualitative data from the structured nurse interviews was coded by two members of the research team. Disagreements were discussed by the coders and resolved. Themes were based on nurses’ experiences implementing specific components of the intervention (e.g., DVD, Tobacco Tactics manual). The qualitative results were triangulated with quantitative results, which involve cross verifying the same information from different sources, in this case comparing patient data, nurse survey and interview data, and EMR data. It was expected that the rich qualitative data obtained from a smaller number of nurse interviews could further explain the more closed-ended, but more generalizable, patient and nurse surveys and EMR data that could be obtained from larger numbers of participants.

## Results

### Reach

#### Patient participation and follow-up rates from recruitment logs, EMR downloads, and patient surveys

Recruitment logs showed that across the three intervention and two control hospitals, 4013 smokers were approached from October 2011 through May 2013. Of these, 2136 were eligible, 608 refused, and 1528 were enrolled (71.5 % participation rate) of which 158 were in the transition period while nurses were being trained and were therefore not included in the analysis. The patient follow-up rates for intervention and control sites were similar at 61.5 % (*n* = 641/1042) and 61.3 % (*n* = 298/486), respectively. Patient surveys and EMR data showed that non-responders to the follow-up survey were more likely to be male (*p* < 0.001), employed (*p* < 0.01), and have a primary diagnosis of mental disorder (*p* = 0.001). See effectiveness paper for recruitment and retention flowchart [34].

#### Patient characteristics from patient surveys and EMR downloads

The description of the sample can be seen in Table 1. The average age was 47.9 years old, just over half were females, and three quarters were white race, and just under one third were married. On average, participants smoked about 15.4 cigarettes per day and 10.0 % used other tobacco products. About one third screened positive for probable problem drinking and one third screened positive for probable depression. Just over one quarter had a diagnosis of unipolar disorder and about one in five had a diagnosis of substance use disorder. The most common discharge diagnoses were diseases of the digestive system (13.1 %), diseases of the circulatory system (13.1 %), injury and poisoning (9.9 %), and diseases of the respiratory system (8.9 %). The most common comorbidities were endocrine, nutritional, and metabolic diseases and immunity disorders (60.2 %), diseases of the circulatory system (53.3 %), mental disorders (50.4 %), diseases of the digestive system (35.5 %), symptoms, signs, and ill-defined conditions (35.4 %), and diseases of the respiratory system (34.3 %).

**Table 1:** Baseline characteristics of the patient sample—*reach* (*n* = 1370)

	Mean	SD
Age (*n* = 1370)	47.9	14.7
Cigarettes per day (*n* = 1364)	15.4	12.0
	*N*	%
Use of other tobacco in past 30 days (*n* = 1368)		
No	1231	90.0
Yes	137	10.0
AUDIT-C (problem drinking) (*n* = 1359)		
<4 for males; <3 for females	892	65.6
≥4 for males; ≥3 for females	467	34.4
PHQ2 (probable depression) (*n* = 1358)		
PHQ < 3	915	67.4
PHQ ≥ 3	443	32.6
Psychiatric comorbidities (*n* = 1370)		
Primary psychotic	35	2.6
Bipolar	134	9.8
Unipolar	384	28.0
PTSD	21	1.5
Substance abuse	264	19.3
Discharge diagnosis from ICD-9 codes (*n* = 1354)		
Infectious and parasitic diseases	96	7.0
Neoplasms	45	3.3
Endocrine, nutritional and metabolic diseases and immunity disorders	66	4.8
Diseases of the blood and blood-forming organs	9	0.7
Mental disorders	96	7.0
Diseases of the nervous system and sense organs	41	3.0
Diseases of the circulatory system	179	13.1
Diseases of the respiratory system	122	8.9
Diseases of the digestive system	180	13.2
Diseases of the genitourinary system	50	3.7
Complications of pregnancy, childbirth, and the puerperium	77	5.6
Diseases of the skin and subcutaneous tissue	56	4.1
Diseases of the musculoskeletal system and connective tissue	95	7.00
Congenital anomalies	0	0.0
Certain conditions originating in the perinatal period	0	0.0
Symptoms, signs, and ill-defined conditions	107	7.8
Injury and poisoning	135	9.9
Procedures	11	0.8
Comorbidities or secondary discharge diagnoses from ICD-9 codes (*n* = 1370)		
Infectious and parasitic diseases	191	13.9
Neoplasms	66	4.8
Endocrine, nutritional and metabolic diseases and immunity disorders	825	60.2
Diseases of the blood and blood-forming organs	315	23.0
Mental disorders	691	50.4
Diseases of the nervous system and sense organs	394	28.8
Diseases of the circulatory system	730	53.5
Diseases of the respiratory system	470	34.3
Diseases of the digestive system	486	35.5
Diseases of the genitourinary system	296	21.6
Complications of pregnancy, childbirth, and the puerperium	75	5.5
Diseases of the skin and subcutaneous tissue	113	8.2
Diseases of the musculoskeletal system and connective tissue	333	24.3
Congenital anomalies	13	0.9
Certain conditions originating in the perinatal period	0	0.0
Symptoms, signs, and ill-defined conditions	485	35.4
Injury and poisoning	254	18.5
Sex (*n* = 1370)		
Male	667	48.7
Female	703	51.3
Ethnicity (*n* = 1370)		
Non-Hispanic	1323	96.6
Hispanic	47	3.4
Race (*n* = 1370)		
White	1051	76.7
African American/Black	237	17.3
Other	82	6.0
Education level (*n* = 1358)		
High school or lower	765	56.3
Some college or higher	593	43.7
Marital status (*n* = 1368)		
Married/domestic partner	435	31.8
Separated/divorced/widowed	490	35.8
Never married	443	32.4
Employment status (*n* = 1365)		
Employed	393	28.8
Unemployed	355	26.0
Retired/disabled/homemaker	617	45.2

#### Self-reported receipt of tobacco services from patient surveys

In the intervention sites, more patients (39.9 %) in the post-intervention period reported receiving handout materials compared to the pre-intervention period (28.4 %) (*p* < 0.001), whereas there was a decrease in receipt of handout materials in the control group pre- to post-intervention (30.2 % pre- versus 20.5 % post-intervention; *p* < 0.01).

### Adoption

#### Description of participating units from recruitment logs and related notes

All targeted medical surgical units and several non-targeted units participated. In one intervention site, obstetrics/gynecology (OB/GYN) nurses requested participation and were given specialized training on tobacco cessation with pregnant smokers and, since there are no evidence-based guidelines for medications with this population, nurses were counseled to work closely with the patient’s obstetrician. Labor and delivery nurses also performed the intervention with expectant fathers, although fathers were not technically patients and therefore could not be given medications and were not followed. This same hospital chose to include the cardiac care unit (CCU), progressive care unit (PCU), and medical detoxification unit but not the psychiatric/behavioral health and cardiovascular and thoracic surgery units, yet nurses on these units requested and were provided with materials. Another intervention site chose to include the CCU. A third intervention site chose to train all outpatient nurses. One control site chose to include the behavioral health/substance abuse unit and another site chose to include the acute rehabilitation unit.

#### Participation in pre-intervention nurse surveys from recruitment logs

Across both intervention and control hospitals, 76.7 % (*n* = 1403/1829) of targeted nurses and 317 non-targeted providers returned pre-intervention surveys, for a total of 1720 participants. Pre-intervention nurse survey response rates were 76.0 % (*n* = 1028/1352) at intervention sites and 78.6 % (*n* = 375/477) at control sites.

#### Characteristics of nurses in pre-intervention surveys

Characteristics of the pre-intervention nurses showed that 37.0 % were less than 35 years old, 45.9 % were 35 to 54 years old, and 17.1 % were greater than 54 years old. Nearly all were female (92.5 %), non-Hispanic (98.6 %), white (89.5 %), and only 4.9 % reported smoking. About 68.3 % had a 4-year degree and 97.6 % were registered nurses (RNs). About 45.1 % worked on medical surgical units, 21.7 % worked on intensive care units (ICU/CCU/PCU), 11.6 % worked on obstetric units, and 21.7 % worked on other units (float, emergency, behavioral health, or outpatient/diagnostics).

#### Participation in and results of nurse post-intervention surveys

Nurse recruitment logs showed that across both intervention and control hospitals, 63.5 % (*n* = 1098/1730) of targeted nurses and 47 non-targeted providers returned 3-month follow-up surveys, for a total of 1145 participants. Post-intervention nurse survey response rates were 62.0 % (*n* = 802/1293) at intervention sites and 67.7 % (*n* = 296/437) at control sites. Nurse surveys showed that characteristics of the post-intervention sample of nurses was similar to the pre-intervention sample with one exception (educational differences) noted below. Note, the number of targeted nurses decreased pre- to post-intervention across intervention and control sites due to staff reductions (1829 pre- to 1730 post-intervention). Additionally, 10.8 % (*n* = 140/1293) of nurses at intervention sites were interviewed.

#### Participation in and opinions about the tobacco tactics training from nurse recruitment logs and surveys

In the intervention hospitals only, recruitment logs showed that 76.0 % (*n* = 1028/1352) of targeted inpatient RN and licensed practical nurses (LPNs) and 317 additional, non-targeted providers employed at the time of intervention participated in the training, for a total of 1345 participants. Nearly all nurses who attended the training, 99.2 % (*n* = 1334/1345), completed surveys immediately after training. About 92.4 % were extremely/somewhat satisfied with the training, 97.9 % rated pharmaceutical management as good/very good/excellent, 97.3 % rated behavioral management as good/very good/excellent, 97.1 % agreed or strongly agreed that they understood it, and 90.9 % thought it was extremely/somewhat helpful.

### Implementation

#### Pre-to post-intervention changes in nurses’ attitudes, behaviors, and barriers to implementing the intervention from surveys

Table 2 shows that nurses in the intervention hospitals reported pre- to post-intervention increases in feeling that providing smoking cessation is important, feeling confident in providing smoking cessation services, and providing smoking cessation services, while there were significant decreases in the control hospitals. Nurses in the intervention hospitals reported increases in providing advice to quit, counseling, medications, handouts, and DVD, while there were no increases in the control hospitals. Moreover, nurses in the intervention hospitals reported significantly decreased barriers to implementing smoking cessation services, while there were marginally significant increases in selected barriers in the control hospitals. To determine reasons as to why the control hospitals actually did worse over time, post hoc analyses were conducted to determine if there were differences in the characteristics of the nurse sample pre- to post-intervention. In the control hospitals only, 62.3 % of nurses reported having at least a 4-year degree pre-intervention, but this dropped to 52.1 % post-intervention (*p* < 0.05), while there was no significant change in the educational preparation of the nurses pre- to post-intervention in the intervention group. On average, intervention site nurses spent about 8 min counseling each smoker.

**Table 2:** Changes in nurses’ self-reported attitudes and behaviors regarding providing cessation services—*implementation*

	Pre-intervention = 1345	Post-intervention = 849	Chi-square
	Control = 375	Control = 296	
	*n* (%)	*n* (%)	*P* value
Smoking cessation is very or extremely important			
Intervention	1015 (75.9)	567 (83.6)	*<0.001*
Control	249 (75.0)	145 (54.9)	*<0.001*
Very or extremely confident in ability to provide smoking cessation			
Intervention	382 (28.6)	387 (57.1)	*<0.001*
Control	141 (43.1)	80 (30.3)	*<0.001*
Currently provide smoking cessation services			
Intervention	1134 (84.9)	635 (92.4)	*<0.001*
Control	273 (82.0)	195 (73.0)	*0.009*
Smoking cessation services provided:			
Advice			
Intervention	940 (83.9)	588 (93.5)	*<0.001*
Control	242 (90.3)	161 (83.9)	*0.039*
Individual counseling			
Intervention	267 (23.8)	255 (40.7)	*<0.001*
Control	91 (34.1)	53 (27.7)	0.150
Group counseling			
Intervention	48 (4.3)	43 (6.9)	*0.019*
Control	21 (7.9)	24 (12.6)	0.095
Medications			
Intervention	845 (75.7)	533 (85.6)	*<0.001*
Control	227 (84.7)	157 (81.8)	0.404
Handouts			
Intervention	1022 (91.1)	593 (94.3)	*0.017*
Control	200 (74.6)	148 (77.1)	0.545
DVD			
Intervention	94 (8.4)	88 (14.0)	*<0.001*
Control	16 (5.9)	13 (6.8)	0.709
Phone calls			
Intervention	35 (3.2)	43 (6.9)	*<0.001*
Control	9 (3.4)	7 (3.7)	0.866
Face barriers that make it difficult to provide smoking cessation services			
Intervention	1042 (78.9)	431 (64.2)	*<0.001*
Control	199 (59.9)	148 (56.1)	0.340
Barriers indicated:			
Lack of confidence			
Intervention	198 (14.7)	74 (8.7)	*<0.001*
Control	24 (6.4)	16 (5.4)	0.589
Not enough training			
Intervention	435 (32.3)	42 (4.9)	*<0.001*
Control	78 (20.8)	67 (22.6)	0.566
Not enough time			
Intervention	665 (49.4)	246 (29.0)	*<0.001*
Control	104 (27.7)	87 (29.4)	0.636
Hesitant to upset patients			
Intervention	378 (28.1)	134 (15.8)	*<0.001*
Control	49 (13.1)	53 (17.9)	0.083
Not my job			
Intervention	50 (3.7)	8 (0.9)	*<0.001*
Control	15 (4.0)	22 (7.4)	0.053

Nurses from non-targeted units were intentionally not surveyed in the post-intervention period, thus the sample size is smaller than the pre-intervention period

#### Implementation of services downloaded from the EMR

To further determine nurses’ charted provision of services in the intervention sites, post-intervention de-identified data was downloaded from the EMR documentation template from all smokers (enrolled in the study or not) during a 5-month period. As shown in Table 3, providing the brochure and Tobacco Tactics manual was charted in over 50 % of identified smokers interested in quitting, while one in five participants was charted to have been given medications.

**Table 3:** Nurses charted documentation on EMR template (*n* = 1388)—*Implementation*

Intervention type	*n*	Percentage of total sample	Percentage of omitting refusers
Refused intervention, given brochure	251	18.1	–
Accepted intervention, given brochure	768	55.3	67.5
Provided Tobacco Tactics manual	578	41.6	50.8
Provided nicotine replacement therapy	252	18.2	22.1
Provided behavioral counseling	243	17.5	21.4
Provided 1-800-QUIT-NOW card	214	15.4	18.8
Provided FDA smoking medication counseling	213	15.3	18.7
Shown DVD	22	1.6	1.9
Provided Bupropion or Chantix	14	1.0	1.2

*n* = 1388 includes de-identified data from all smokers including those enrolled and not enrolled in the study during 5-month time period

#### Implementation of services from nurse interviews

Of the 140 targeted nurses interviewed 2 to 6 months post-intervention at the intervention sites only, over 82.9 % reported implementing all components of the intervention, except only 53.6 % reported showing the DVD [44]. Qualitative comments from the nurse interviews shown in Table 4 indicate that nurses did not show the DVD largely because the overhead television system was not working or when it was working, it was cumbersome to use. Moreover, the nurses reported that charting was problematic as it took some time to integrate the documentation template into the EMR. Overall, the qualitative comments were very positive using words such as “user friendly,” “streamlined,” or “saves time” to describe the program and materials.

**Table 4:** Qualitative information provided during interviews) – *Implementation*

Component	Comments
Brochure	Put brochure in patient’s admission package “I handed the brochure to the partner of a patient.” “I don’t always provide the brochure. It is sometimes inconvenient.”
DVD	Patients are usually not interested “Accessing the DVD is too difficult for patients”
Tobacco Tactics manual	“I really like it.” (5) “User friendly.” “Materials make it easy; it’s simple enough for patients with less education.” “The book saves me a lot of time talking.” “The book is informative and simple, that is good.” “The manual is kind of long and wordy.”
Tobacco Tactics training	“I have to admit that I had an attitude when I went to the training, but it was much more interesting than I thought.” “I liked the training. I feel more comfortable talking to patients now” “The training gave me more ideas.” “I complement the program and planning. It is presented well and attainable.” “The training made me more comfortable talking to patients.” “I like that I can take this information and apply it to other issues as well.” “Training was mandatory on our unit.” “I did not attend the training, but learned the content from other nurses who went.” “I was too busy at the time to attend training.” (2)
Tobacco Tactics intervention	“This [Tobacco Tactics intervention] is more than we had before.” “The intervention is more streamlined now.” “This is a good service, the patients appreciate it; it’s just that we’re too busy with other things, so we don’t always remember to address smoking.” “I give materials to fathers too.” (nurse on OB/GYN unit)
Charting	“Charting is a problem” (11)
What nurses say to patients	“Quitting will improve wound healing and overall health.” (4) “I say you haven’t smoked this long, it’s a good time to quit.” “The [quit] day is here.” “Never quit quitting!” “Smoking is the #1 modifiable risk factor.” “I focus on support group and home.” “Don’t smoke in front of children.” Asks if spouse smokes. “I encourage patients to write down how much money they safe each time they don’t smoke for a day.” “I say: ‘Either it is gas money, or it is cigarette money.’” “I tell them: ‘direct your brain to s.th. [something] else.’” From an email: … “just a few short hours after I was in your tobacco tactics class on Monday… I was taking care of a patient who smoked a pack a day and had wanted to quit for a long time. I sat down with her and told her how important it was for her health to quit smoking (yes, I used the phrase)! She agreed and said that she really wanted to quit but that she wasn’t ready because she needed help. I reminded her that she wasn’t going to be smoking while she was here (we had her on a nicotine patch) and that now would be a great time to quit. She agreed and said how she has her son’s wedding coming up in December and she would love to be smoke-free by then. I explained how that would be an excellent goal and that she might as well start now! She looked at me, smiled, and said, “let’s do it!” I gave her the Tobacco Tactics book and the 1-800-quit now card and she spent the rest of the evening looking through the book! She told the day shift RN during bedside report that I had convinced her to quit smoking and that she was going to stick with it! YAY!”
Cessation help strategies	“I provide counseling only if patients want to.” “It is important to keep reminding patients when they are in the hospital, since it is important for their health.” “Education is the biggest thing; I point out the benefits and provide examples from the patient’s life, e.g., I relate quitting to the grandchildren.” “Almost always gives support, but not always the brochure, will now.” “A patient with lumbar fusion will be offered Bupropion or Chantix.” “We should frame it in the baby’s health framework.” “Removing cigarettes from home and work is a good implementation strategy for the patient.”
Opinions on smoking and quitting	“Nicotine seems to be more addictive nowadays.” “Patients don’t mind to hear about quitting from me.” “Patients with lung issues tend to want to quit.” “Smoking cessation is like one other thing, something that gets glazed over. I do stress cessation with vascular patients.” “If they don’t want to quit, I still provide additional counseling.” “I’m impressed that there is a phone number that they can call.” “It’s patients with a lower SES who smoke more.” “I think we have fewer smokers than we used to.” “Quitting is nothing more than a decision. It’s more psychological than anything.” “Patients quit at their own time, when they are ready, I don’t push it.” “Most people don’t want to hear anything about quitting.” “We have a lot of Alzheimer’s patients. They are too confused for the intervention.” “Our patients often smoked a long time ago.” “I’m not uncomfortable talking to patients.”

#### Implementation of follow-up calls from volunteer telephone logs

At the 3 intervention sites, volunteers made at least 1057 attempts/phone calls to 228 patients over a 5-month period in 2013, of which 63.2 % were reached at least once. An average of 2.2 follow-up phone calls per patient were completed to 144 patients, for a total of 313 patient contacts. The remaining attempts/phone calls resulted in voicemail messages, no answers, speaking with someone other than the patient, and unreachable phone numbers.

### Maintenance/sustainability

To enhance sustainability, training was incorporated into new hire nurse training at all sites. The information in Tables 2, 3, and 4, which was collected during the post-intervention period, suggests that there was short-term sustainability. While data were not systematically collected on long-term sustainability of the program, there is anecdotal evidence that the program is being maintained, as members of the study team are periodically contacted by Trinity Health nurses with questions, primarily regarding where to order more materials. One of the intervention hospitals reported that they changed the nurse training from face-to-face to online training. Two other Trinity Health hospitals heard about the program, contacted us, and are implementing components of the intervention.

## Discussion

### Reach

The intervention had high reach as demonstrated by post-intervention patients in the intervention sites reporting receiving significantly more handout materials. Unlike telephone quit lines, which have been shown to be highly effective, but reach only 6 to 10 % of smokers [45], numerous studies have shown that inpatient cessation programs, including those delivered by nurses [2, 46], have the potential to reach a large number of captive smokers [47–50]. Participants in the sample had many characteristics that placed them at high risk for smoking and relapse back to smoking [51] including probable problem drinking, depression, and less than a high school education [52]. Diseases of the respiratory and circulatory systems were among the most common and are often smoking-related [53, 54], which may increase motivation to quit [55].

### Adoption

One of our best measures of success is that over three quarters of targeted nurses participated in the Tobacco Tactics training on targeted units with additional participation from non-targeted units. The high satisfaction with the training was likely discussed among nurses and their managers resulting in many nurses attending from non-targeted units. The extension of the intervention to various types of intensive care units, outpatient, emergency room, psychiatric, substance abuse, and obstetric units (including expectant fathers) speaks not only to the to the quality of the program but also for the need for training providers to conduct tobacco cessation interventions in hospital settings.

Nurses increased perceived importance of and self-confidence in delivering tobacco cessation services likely contributed to the increased adoption of the intervention and subsequent increased quit rates in the intervention sites. Our prior work has shown that nurses who were more satisfied with and had a better understanding of the Tobacco Tactics intervention had significantly higher perceived confidence scores and importance scores related to providing the intervention [56]. Providers who feel more confident in their ability to do what is expected, recognize the need and importance for the intervention, and have the requisite skills are more likely to implement a program at higher levels of fidelity and result in increased quit rates [57–59].

### Implementation

With as little as a 1-h training session, the proportion of nurses self-reporting the provision of cessation services significantly increased from pre- to post-intervention. This finding is congruent with a Cochrane Collaborative Review that demonstrated that health professional training increases the delivery of smoking cessation interventions [59]. Implementing standard protocols, which have been shown to be effective in increasing smoking cessation counseling interventions provided by nurses [60], likely enhanced service delivery [61]. Nurses are ideally positioned to deliver cessation interventions because (1) physician time is at a premium, (2) nurses are educated in psychosocial and physiological interventions, (3) nurses have access to and immediate rapport with patients as well as respect from physicians, (4) nurses understand the patient’s medical condition and can tailor the intervention accordingly, and (5) nurses can read charts, initiate medication orders, and write nursing notes.

It is interesting to note that in the control group, nurses pre- to post-intervention self-reported a decrease in feeling that providing smoking cessation is important, a decrease in feeling confident in providing smoking cessation services, and less provision of smoking cessation services, and selected barriers increased at marginally significant levels. Moreover, patients in the control group reported receiving less smoking cessation handouts post-intervention compared to pre-intervention. This may be related to the lower educational levels of the nurses in the post-intervention period compared to the pre-intervention period. This is similar to another smoking cessation implementation study that also trained nurses and found that low performing units had nurses that were less educationally prepared [50].

Lack of time and competing demands have been cited as barriers to providing cessation services by nurses in other studies [62]. Yet, our work and the work of others [63, 64] have shown that the major barrier to nurses providing cessation services is not lack of time but lack of expertise, as the nurses in our current study reported that the intervention “saves time.” Although in our prior VA study [20], splicing the DVD into the overhead television system was easy and nurses reported that showing the patient the DVD saved them time at the bedside, the DVD was not easily integrated into the overhead television system used by Trinity Health.

While several studies [65, 66] and this study report the use of the EMR to identify smokers, there are few studies [19] that use the EMR to actually document the treatment of smoking in accordance with JC standards [38]. While qualitative comments supported the need for improvement in the documentation template, there is motivation for hospital leadership to conduct this programming as meaningful use (the set of standards defined by the Centers for Medicare & Medicaid Services Incentive Programs that governs the use of EMRs) will allow eligible providers and hospitals to earn incentive payments for implementing tobacco cessation strategies [67]. Currently, the JC is starting to work with Mathematica Policy Research and the Center for Medicare and Medicaid Services to assemble the Tobacco Treatment Task Force, which will reengineer these measures as electronic clinical quality measures.

### Maintenance

The nurse-delivered Tobacco Tactics program remained sustainable 1 year after the researchers withdrew from the settings and longer term sustainability has been anecdotally verified. Integrating the program into new nurse training has enhanced sustainability as nurses turn over. While volunteers made phone calls during the study, unfortunately, there was no one from any of the intervention hospitals invested in coordinating the follow-up phone calls when the study ended. This is similar to our prior VA study that implemented and evaluated volunteer follow-up phone calls for smoking cessation and found it to be effective and cost-effective yet was not sustainable for similar reasons [68].

### Application of the RE-AIM framework to assess intervention implementation

This study has shown how the RE-AIM framework can be used to guide research-based interventions in clinical practice. Utilization of the RE-AIM framework can serve as a guide to plan, conduct, and report on interventions that are implemented on a large scale in real-world settings [16]. Not only can the RE-AIM framework identify individual impact, but it can also identify population impact, as was done in this study [69]. The framework can be used to maximize external validity; report elements of both internal and external validity; review a body of evidence; and compare interventions to make policy decisions [23, 24, 70–73].

### Strengths and limitations of the study

While pragmatic designs are more feasible and allow for implementation in natural environments [74], the lack of randomization may pose challenges in terms of internal validity, making it harder to rule out confounding variables [75]. Although data from one of the control hospitals did not materialize, the nurse and patient sample sizes in the other two control hospitals were large enough to allow clinically and statistically meaningful comparisons with the intervention hospitals. Nurses’ implementation of the Tobacco Tactics intervention was based on self-report and therefore may have been inflated, although there was no inflation in the nurse self-report in the control group. Moreover, self-reported implementation of the Tobacco Tactics intervention was somewhat substantiated by EMR-downloaded documentation of services and qualitative comments by nurses reflected a large amount of detail about and enthusiasm for implementing the intervention.

## Conclusions

The Tobacco Tactics intervention had high *reach* among inpatient smokers. *Adoption* and *implementation* were also high probably due to the packaging of the intervention into a user-friendly toolkit. Short-term maintenance was substantiated by patient and nurse surveys, and anecdotal evidence suggests that the program remains sustainable. Imagine the enormous reach and public health impact of an inpatient smoking cessation intervention if the largest group of front-line providers, namely nurses, were trained to effectively provide the tobacco cessation interventions. As we move toward more population-based interventions, the RE-AIM framework is a valuable guide for implementation.

## Additional files

Additional file 1: Summary of RE-AIM measures. (DOCX 22 kb)
Additional file 2: Staff—3-month post-training survey. (DOCX 79 kb)
Additional file 3: Nurse interview guide. (DOCX 17 kb)
