# Supplementary material for: Implementation of the Tobacco Tactics intervention versus usual care in Trinity Health community hospitals
Source: Implement Sci. 2016 Nov 4;11:147. doi: 10.1186/s13012-016-0511-6 (PMC5097410; doi:10.1186/s13012-016-0511-6)
Supplement: Additional file 1: — Summary of RE-AIM measures. (DOCX 22 kb) [file 13012_2016_511_MOESM1_ESM.docx]

**Additional File 1. Summary of RE-AIM Measures**

| **RE-AIM Constructs** | **Measurement Tool** | **Description of Measure** |
| --- | --- | --- |
| **Reach:** percent and representativeness of individuals receiving an intervention. | - Patient participation and follow-up rate from recruitment logs. - Patient surveys/EMR data. | - Number enrolled/number eligible. - Characteristics of patient sample. - Self-reported receipt of smoking cessation print materials. |
| **Effectiveness** (previously published, hence not presented in this paper): impact of an intervention on outcomes. | - Patient surveys. - Patient NicAlert tests. | - Self-reported 6-month smoking quit rates. - Cotinine verified 6-month-day smoking quit rates. |
| **Adoption:** proportion and representativeness of settings and providers willing to deliver the intervention. | - Description of participating units from recruitment logs. - Pre- and post-intervention nurse survey participation and follow-up rates from recruitment logs. - Characteristics of nurses from surveys. - Nurse participation rate in training from recruitment logs. - Opinions about training from surveys. | - Number survey responders/number eligible. - Characteristics of nurse sample. - Number of targeted nurses trained/number eligible. - Number of non-targeted staff trained.   *Opinions from Surveys*   - Overall satisfaction with training. - Satisfaction with pharmaceutical management. - Satisfaction with behavioral management. - Understanding of training. - Helpfulness of training. |
| **Implementation:** extent to which the intervention is implemented as intended. | - Nurse surveys. - Download of documentation from EMR. - Nurse interviews. - Number of volunteer follow up calls from telephone logs. | - Pre- and post-intervention nurse self-reported attitudes, delivery of services, and barriers to delivering smoking cessation services from surveys. - Percent documentation of specific components of the intervention. - Post-Intervention interview-reported quantitative and qualitative data on delivery of specific components of the intervention from interviews. - Number volunteer telephone follow-up attempts, percent reached, average number of calls per patient, number of patients reached, and total number of contacts. |
| **Maintenance (short and long term):** sustainability of an intervention at individual and setting levels. | - Short-term: Patient follow-up surveys in post-intervention period. - Short-term: Nurse follow-up surveys in post-intervention period. - Long-term: Anecdotal communication with facilities. | - Patient reported receipt of services in follow up period. - Nurses reported delivery of services in follow up period. - Nurse training incorporated into new nurse orientation. - Trinity Health nurses continue to ask smoking cessation questions and order additional Tobacco Tactics materials after study conclusion. |
