# Supplementary material for: Implementation of the Tobacco Tactics intervention versus usual care in Trinity Health community hospitals
Source: Implement Sci. 2016 Nov 4;11:147. doi: 10.1186/s13012-016-0511-6 (PMC5097410; doi:10.1186/s13012-016-0511-6)
Supplement: Additional file 2: — Staff—3-month post-training survey. (DOCX 79 kb) [file 13012_2016_511_MOESM2_ESM.docx]

**Additional File 2. Staff - 3-Month Post-Training Survey.**

|  |
| --- |
| **Staff – 3 Month Post-Training Survey** |
|  |

**Tobacco Tactics**

**for Hospitalized Smokers**

We are evaluating the training you may have received to provide smoking cessation services to patients at Saint Mary’s Health Care. The information you provide on this survey will help us evaluate knowledge about smoking cessation interventions. Completing the survey should take about 5 to 10 minutes.

Participation in this survey is completely voluntary. You may choose not to answer any question that makes you feel uncomfortable.

Please do NOT put your name on the survey as all of your answers will be kept anonymous and confidential. Only study researchers will have access to the information on this survey.

Your answers to the questions are very important to us. Thank you for agreeing to complete this brief smoking survey. Please return the completed survey in the attached envelope.

Sincerely,

Christine Werkema, MS, RN, OCN, AOCN

Sonia A. Duffy, PhD, RN


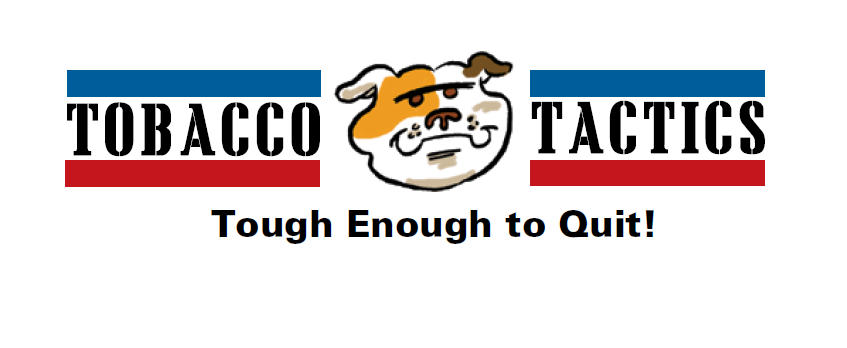


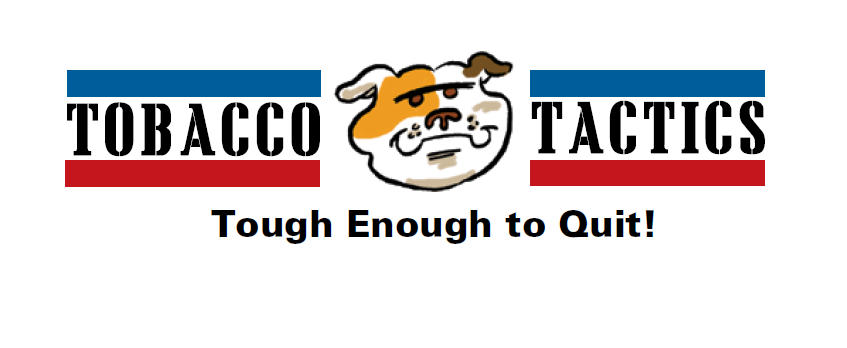


## Smoking Cessation

**3-Month Post-Training Staff Survey**

***The following questions ask about the training you may have received to provide smoking cessation services some time ago. Now that you have had a chance to apply the training for use with your patients, please answer each of the following questions by circling the answer. If you are unsure about how to answer a question, please give the best answer you can.***

1. Did you participate in the training for smoking cessation?

NO……………..………..….. □

YES…………………….…... □

***If yes, please continue to Q2.***

***If no, please indicate the reason for not attending the training and return the survey.***

***__________________________________________________________________***

***__________________________________________________________________***

1. ***If yes***, in general how satisfied were you with the material presented?

| **Extremely Not Satisfied** | **Somewhat Not Satisfied** | **Neutral/ Undecided** | **Somewhat Satisfied** | **Extremely Satisfied** |
| --- | --- | --- | --- | --- |
| 1 | 2 | 3 | 4 | 5 |

1. How well would you rate the following training sessions?

|  | **Poor** | **Fair** | **Good** | **Very Good** | **Excellent** | **Not Applicable** |
| --- | --- | --- | --- | --- | --- | --- |
| **Pharmaceutical Management** | 1 | 2 | 3 | 4 | 5 | 6 |
| **Behavioral Management** | 1 | 2 | 3 | 4 | 5 | 6 |

1. Do you feel you have a good understanding of the elements of the Smoking Cessation Intervention?

| **Strongly Disagree** | **Disagree** | **Neutral** | **Agree** | **Strongly Agree** |
| --- | --- | --- | --- | --- |
| 1 | 2 | 3 | 4 | 5 |

1. How important do you think it is to provide smoking cessation services to smokers?

| **Not At All Important** | **Somewhat Important** | **Moderately Important** | **Very Important** | **Extremely Important** |
| --- | --- | --- | --- | --- |
| 1 | 2 | 3 | 4 | 5 |

1. How confident are you in your abilities to provide smoking cessation services to smokers?

| **Not At All Confident** | **Somewhat Confident** | **Moderately Confident** | **Very Confident** | **Extremely Confident** |
| --- | --- | --- | --- | --- |
| 1 | 2 | 3 | 4 | 5 |

1. How helpful do you think the smoking cessation program you received training on is to smokers?

| **Extremely Unhelpful** | **Somewhat Unhelpful** | **Neutral/ Undecided** | **Somewhat Helpful** | **Extremely Helpful** |
| --- | --- | --- | --- | --- |
| 1 | 2 | 3 | 4 | 5 |

1. Prior to receiving the training on smoking cessation, did you personally provide smoking cessation services to smokers?

NO……………..………..….. □

YES…………………….…... □

| 1. Do you currently provide smoking cessation services to smokers? |
| --- |

NO……………..………..….. □

YES…………………….…... □

***If yes, continue with Q10.***

***If no, please indicate why you do not provide these services:***

Lack of confidence …..…………… □

Not enough training ……………...... □

Not enough time …………….…...... □

Hesitant to upset patients ………… □

Not my job ………………….…....... □

Other ……………………………..... □

***Please specify*** __________________________________________________________________

__________________________________________________________________

1. ***If yes***, which of the following services do you personally provide or initiate?

|  | **No** | **Yes** |
| --- | --- | --- |
| - 1. Advice to stop using tobacco products | 0 | 1 |
| - 1. Individual counseling for quitting smoking | 0 | 1 |
| - 1. Group counseling for quitting smoking | 0 | 1 |
| - 1. Medications (for example, the patch) to help patients quit smoking | 0 | 1 |
| - 1. Hand-out materials (pamphlet or workbook) to help patients quit smoking | 0 | 1 |
| - 1. Video about quitting smoking | 0 | 1 |
| - 1. Phone calls about quitting smoking after patients are discharged | 0 | 1 |

1. On average, how many minutes total do you spend counseling the average smoker?

_____________________ MINUTES

1. Are there any barriers which make it difficult to provide smoking cessation services to smokers?

NO……………..………..….. □

YES…………………….…... □

***If yes, please indicate the barriers below:***

Lack of confidence …..…………… □

Not enough training ……………...... □

Not enough time …………….…...... □

Hesitant to upset patients ………… □

Not my job ………………….…....... □

Other ……………………………..... □

***Please specify*** __________________________________________________________________

__________________________________________________________________

__________________________________________________________________

1. Is there anything else your hospital could do to improve the provision of smoking cessation services to smokers?

________________________________________________________________________________________________________________________________________________________________________________________________________________________________________________________________________________________________

***Finally, we would like to know a few things about you. Please provide the correct answer.***

1. Please circle the answer that best describes your smoking status.

I CURRENTLY SMOKE .……… ……………………………………………………..... □

I have smoked in the past, but quit within the last 1 month .…..... □

I have smoked in the past, but quit within the last 6 months .…... □

I have smoked in the past, but quit within the last year .……….... □

I have smoked in the past, but quit over a year ago .………….…..... □

I have never smoked .…… ……………………………………………………..... □

***If you have not smoked cigarettes within the last month, please skip to Q17.***

1. How old are you?

Less than 35 Years………………..... □

35-44 Years……….……………....... □

45-54 Years…………………..…...... □

55-64 Years….……………...…….... □

Greater than 64 Years….................... □

1. What is your sex?

MALE……………..……….. □

FEMALE…………………... □

1. What is your race/ethnicity?

EUROPEAN AMERICAN/WHITE…..……………….................... □

AFRICAN AMERICAN/BLACK……….…………………..…...... □

HISPANIC/LATINO…………......................................................... □

ASIAN/PACIFIC ISLANDER…………..…………..……….......... □

AMERICAN INDIAN/ESKIMO/ALEUTIAN………..................... □

OTHER ……………………………................................................ □

***Please specify*** __________________________________________________________________

1. Do you have a 4-year college degree?

NO……………..………..….. □

YES…………………….…... □

1. How many cigarettes/day do you smoke?

10 or LESS………………..... □

11-20…………….………..... □

21-30……………………...... □

31 or MORE....……...…….... □

1. What is your current position?

RN………………………………………………………………………………….. □

LPN/NURSING ASSISTANT……………………………………………...……... □

OTHER PROFESSIONAL (E.G. SOCIAL WORKER,

RESPIRATORY THERAPIST, PHARMACIST, PHYSICIAN) …………...….. □

1. What unit do you work on? ____________________________________
2. What date did you complete this survey? Date: _____ / _____ / _____

***Thank you so much for participating in the study!***

**Please return the survey in the envelope provided and give it to your unit manager.**

***Please contact Chris Werkema at (XXX) XXX-XXX***

***if you have any questions.***
