# Supplementary material for: Implementation of the Tobacco Tactics intervention versus usual care in Trinity Health community hospitals
Source: Implement Sci. 2016 Nov 4;11:147. doi: 10.1186/s13012-016-0511-6 (PMC5097410; doi:10.1186/s13012-016-0511-6)
Supplement: Additional file 3: — Nurse interview guide. (DOCX 17 kb) [file 13012_2016_511_MOESM3_ESM.docx]

**Additional File 3. Nurse Interview Guide.**

**Nurse Interview Guide**

| **DURING HOSPITAL STAY:** | | |
| --- | --- | --- |
|  | Yes | No |
| **Patient was asked about tobacco use.** | **** | **** |
| **Smoking status was documented.** | **** | **** |
| *Comments:* **____________________________________________________________________________________________________________________________________________________________________________________________________________________________________________________________** |  |  |
| *Prompts:* *Can you tell me more about that? What works? What does not work?*  For patients, who are current tobacco users OR quit within the last 12 months: | | |
|  | Yes | No |
| **PATIENT WAS OFFERED COUNSELING DURING HOSPITAL STAY.** | **** | **** |
| Quit Smoking brochure was provided. |  |  |
| **PATIENT RECEIVED COUNSELING.** | **** | **** |
| **Comments: ____________________________________________________________________________________________________________________________________________________________________________________________________________________________________________________________** |  |  |

*Prompts:* *Can you tell me more about that? What works? What does not work?*

| Behavioral component | | |
| --- | --- | --- |
|  | Yes | No |
| Advised the patient to set a quit date, ideally within 2 weeks |  |  |
| Advised the patient to remove all tobacco products from the home and at work. |  |  |
| Discussed potential challenges to quitting, staying off tobacco, and planning ahead on how to deal with the challenges. |  |  |
| Provided the patient with strong messages of support and encouragement. |  |  |
| Stop Smoking videotape was provided. |  |  |
| Tobacco Tactics workbook was provided. |  |  |
| **Information about FDA-approved cessation medication was provided.** | **** | **** |
| **Patient received prescription for FDA-approved cessation medication.** | **** | **** |

**Comments: ______________________________________________________ ___________________________________________________________________________________________________________________________________________________________________________________________________**

| *Prompts:* *Can you tell me more about that? What works? What does not work?*  **AT DISCHARGE:** | | |
| --- | --- | --- |
|  | Yes | No |
| **Patient was offered referral to outpatient counseling.** |  |  |
| **Patient received referral to outpatient counseling.** |  |  |
| **Patient was offered prescription for FDA-approved cessation medication.** |  |  |
| **Patient received prescription for FDA-approved cessation medication.** |  |  |

Comments:**_______________________________________________________________________________________________________________________________________________________________________________________________________________________________**

*Prompts:* *Can you tell me more about that? What works? What does not work?*

*Are there any other comments that you would like to make About the Tobacco Tactics Intervention?*

**__________________________________________________________________________________________________________________________________________________________________________________________________________________________________________________________________________________________________________________________________________________________________________________________________________________________________________________________________________________________________________________________________________________________________**

*Prompts:* *Can you tell me more about that? What works? What does not work?*

**Bolded items are Joint Commission standards.**
